# Harmonizing Quality Improvement Metrics Across Global Trial Networks to Advance Paediatric Clinical Trials Delivery

**DOI:** 10.1007/s43441-024-00663-0

**Published:** 2024-06-20

**Authors:** Sabah Attar, Angie Price, Collin Hovinga, Breanne Stewart, Thierry Lacaze-Masmonteil, Fedele Bonifazi, Mark A. Turner, Ricardo M. Fernandes

**Affiliations:** 1 Department of Women’s and Children’s Health, Liverpool Women’s NHS Foundation Trust, University of Liverpool, Crown Street, Liverpool, L8 7SS UK; 2 conect4children Stichting, Utrecht, The Netherlands; 3 Site Network - Quality Improvement, Institute for Advanced Clinical Trials for Children, 9200 Corporate Blvd, Suite 350, Rockville, MD 20850 USA; 4 Clinical and Scientific Development, Institute for Advanced Clinical Trials for Children, 9200 Corporate Blvd, Suite 350, Rockville, MD 20850 USA; 5 Clinical Trials Office, College of Health Sciences, Faculty of Medicine and Dentistry, University of Alberta, 400 College Plaza 8215 - 112 Street, Edmonton, AB T6G 2C8 Canada; 6 Maternal Infant Child Youth Research Network, Vancouver, BC Canada; 7 Department of Pediatrics, Cumming School of Medicine, University of Calgary, 3330 Hospital Dr NW, Calgary, AB T2N 4N1 Canada; 8 Fondazione per la Ricerca Farmacologica Gianni Benzi Onlus, Via Abate Eustasio, 30, 70010 Valenzano, BA Italy; 9 Institute of Life Course and Medical Sciences, Liverpool Women’s NHS Foundation Trust, University of Liverpool, Crown Street, Liverpool, L8 7SS UK; 10 Liverpool Health Partners, Liverpool, UK; 11 Laboratory of Clinical Pharmacology and Therapeutics, Faculty of Medicine, University of Lisbon, Av. Prof. Egas Moniz MB, 1649-028 Lisbon, Portugal

**Keywords:** Paediatrics, Clinical trials, Networks, Metrics, Quality, Performance

## Abstract

**Background:**

Despite global efforts to improve paediatric clinical trials, significant delays continue in paediatric drug approvals. Collaboration between research networks is needed to address these delays. This paper is a first step to promote interoperability between paediatric networks from different jurisdictions by comparing drivers for, and content of, metrics about clinical trial conduct.

**Methods:**

Three paediatric networks, Institute for Advanced Clinical Trials for Children, the Maternal Infant Child and Youth Research Network and conect4children, have each developed metrics to address delays and create efficiencies. We identified the methodology by which each network identified metrics, described the metrics of each network, and mapped consistency to come to consensus about core metrics that networks could share.

**Results:**

Metric selection was driven by site quality improvement in one network (11 metrics), by network performance in one network (13 metrics), and by both in one network (five metrics). The domains of metrics were research capacity/capability, site identification/feasibility, trial start-up, and recruitment/enrolment. The network driven by site quality improvement did not have indicators for capacity/capability or identification/feasibility. Fifteen metrics for trial start up and conduct were identified. Metrics related to site approvals were found in all three networks. The themes for metrics can inform the development of ‘shared’ metrics.

**Conclusion:**

We found disparity in drivers, methodology and metrics. Tackling this disparity will result in a unified approach to addressing delays in paediatric drug approvals. Collaborative work to define inter-operable metrics globally is outlined.

## Introduction

Significant challenges remain in how paediatric clinical trials are conducted: upto 19% of trials have been reported to discontinue, with up to 38% of trials reporting patient recruitment as the main reason [1, 2]. These results are attributed to issues with the design and operational execution of these trials, including lengthy study start-up times, inability to meet target enrolment goals and poor patient retention rates [1, 2]. The clinical research enterprise needs to transform involving collaboration among diverse public and private stakeholders, innovative re-engineering of the current delivery of clinical trials, and novel methodologies to integrate existing expertise, resources, and infrastructure [3, 4]. These challenges apply to paediatric trials [5–7].

Clinical trial networks can support optimizing trial delivery by implementing quality and performance metrics in alignment with sponsors and sites [8]. Adopting rigorous, harmonized systems and procedures to capture operational metrics and compare them with performance targets can support tracking, evaluating, benchmarking and predicting performance. Metrics should measure the right factors accurately, with standard definitions and data points, to provide actionable information to support planning and decisions [9–11]. Metrics can be used to track outcomes, processes, and performance in clinical trial delivery by sponsors and research networks [9, 12] and can focus on the individual site and protocol levels but also at a portfolio level, across trials and sites [13, 14].

The objectives of this paper are to:

- Describe approaches used by contributing networks to identify and develop key metrics/indicators
- Describe common metrics and challenges in identifying network metrics
- Identify a preliminary set of interoperable metrics

## Methods

An international quality initiative “think tank” was convened with representatives from three paediatric trial networks from different jurisdictions that focus on novel drugs. These networks are specialty-agnostic with wide geographical coverage and work with the Pharmaceutical industry and academic Sponsors. This group, derived from the ongoing dialogue and collaboration between these networks, focused on improving the pediatric research enterprise and infrastructure. The group met remotely from 2021 to 2023 with at least quarterly meetings. Open discussions were driven by sharing of approaches, processes, documents, and experiences from each network.

Metrics and their methods of collections were identified through discussion and sharing within the think tank. The metrics were identified by a survey of the networks. Sources of alignment and divergence and opportunities for shared metrics were identified by consensus between members of the think tank. This work used process data from the networks excluding personal data. Accordingly, review by research ethics committees or Institutional Review Boards was not needed.

### Contributing Networks

#### Institute for Advanced Clinical Trials (I-ACT) for Children

I-ACT was created by a consortium of key stakeholders in paediatrics, including the Critical Path Institute, the American Academy of Pediatrics and others in academia, industry, and the regulatory world. I-ACT is a 501c3 non-profit organization with a mission to serve as a neutral and independent organization on behalf of children everywhere. I-ACT is designed to advance innovative medicines and device development and labelling to improve child health [15]. I-ACT engages public and private stakeholders through research and education to ensure that healthcare for children is continually improved by enhancing the awareness, quality, and support for paediatric clinical trials.

I-ACT currently includes 74 U.S. and international network sites committed to performing paediatric research to support regulatory approval by industry and academic Sponsors. I-ACT supports the network sites by providing clinical trial opportunities, a peer-to-peer mentoring program, educational webinars, professional education grants, and supports sites to improve paediatric research conduct. I-ACT launched the Pediatric Improvement Collaborative for Clinical Trials & Research (PICTR***®***), a quality improvement program to help identify and mitigate the challenges sites face when conducting paediatric clinical trials. The PICTR program collects and analyses paediatric clinical trial operations data at site level to determine best practices and process improvement. The data is shared across the site network. This exchange creates a continuous learning environment to maximize trial speed, quality and efficiency.

#### conect4children (c4c)-Collaborative Network for European Clinical Trials for Children

C4c is an action under the Innovative Medicines Initiative 2 (IMI2) Joint Understanding, Grant Agreement 777389 from 2018 to 2024 [16, 17]. The c4c consortium includes 10 large pharmaceutical companies and 37 non-industry partners, including academia, hospitals, third-sector organizations and patient advocacy groups. The consortium aims to set up and evaluate a pan-European paediatric-focused clinical trial infrastructure tailored to meet the needs of children involved in clinical trials. c4c is focused on four main areas of services, including: strategic feasibility expert advice on study design and/or paediatric development programmes, including patient/parent involvement; a network of over 250 clinical sites across 21 European countries coordinated by 20 National Hubs and a central Network Infrastructure Office, with local knowledge and expertise and aligned processes across the entire network; a Training Academy providing standardized training to all study sites and site personnel; and a Data focused work package to support management of data and metrics used by the network and the development of a standardised paediatric data dictionary. A new legal entity, the conect4children Stichting has been established to ensure sustainability of this project’s results.

#### Maternal Infant Child and Youth Research Network (MICYRN)

MICYRN is a Canadian federal not-for-profit, charitable organization founded in 2006 to build capacity for high-quality applied health research. MICYRN is governed by a Board comprising member research organizations and members at large, who represent specific research foci and expertise. Oversight of the network is maintained through an executive team consisting of the Board chair, vice-chair, scientific directors, and executive directors. The network formally links 21 maternal and child health research member organizations based at academic health centres in Canada; is affiliated with more than 25 practice-based research networks; provides support to new and emerging teams; and has established strong national and international partnerships such as I-ACT and c4c.

The mission of MICYRN is to catalyze advances in maternal and child healthcare by connecting minds and removing barriers to high-quality health research. MICYRN is working towards building a national infrastructure to attract and facilitate the conduct of maternal-child investigator-initiated and industry-sponsored multicenter clinical trials and functions as a de-centralized Academic Research Organization. MICYRN prioritizes quality improvement initiatives, supports training and mentorship programs for emerging investigators and new trainees, and leverages national partnerships to lead advocacy initiatives for regulatory and ethical pathways in Canada. In collaboration with I-ACT, MICYRN is working on a Quality Improvement and Performance Metrics Initiative to collect information on key indicators to improve maternal/child health in Canada.

## Results

### Approaches Used by Contributing Networks to Identify and Develop Key Metrics/Indicators

#### Pediatric Improvement Collaborative for Clinical Research and Trials (PICTR®)

In 2018, PICTR worked closely with members of the site network to assess current paediatric clinical trial research operations. Sites completed surveys about their operations and met frequently to share gaps in their processes. Based on site feedback and subject matter experts (SME), a preliminary list of measurable goals and metrics was developed for improving the clinical trials process within sites.

To ensure the program’s goals and metrics aligned across the industry, PICTR hosted an SME meeting in Chicago in 2019, bringing together key stakeholders to discuss the conduct of clinical trials including pharmaceutical companies, federal agencies, academia, research sites, other global paediatric networks, and patients and families. The meeting outcome was a draft set of six metrics used to identify gaps in the clinical research operations process at site level.

Following the SME meeting, 14 sites participated in a pilot project collecting research operations metrics focused on the institutional review board and contracts process. The pilot aided in validating the program goals and identifying additional metrics after which, there was an ongoing collaboration with key stakeholders resulting in a final set of 11 core research operation metrics (Appendix A). Quality Improvement initiatives for sites were based on these metrics.

#### connect4children-Collaborative Network for European Clinical Trial for Children

C4c collects metrics to measure quality and performance of processes and network. Implementing a Performance Measurement System has a positive organisational effect, improves results over the long term, drives organisational strategy, supports planning and decision-making, and acts as an effective tool for communicating achieved results to stakeholders [18]

Within c4c, a methodological model was developed to identify a list of metrics and underlying data points to be suggested for adoption by c4c. The model considered metrics-specific issues, including:

1. Terminology.
2. Common practice and use of metrics—collected from examples of national networks and sponsors.
3. Lean Management approach in clinical research (e.g. “time” as one of the key performance measures).
4. Goal-Question-Metric Paradigm (defining goals behind the processes to be measured and using these to decide precisely what to measure).
5. Multi-Criteria Decision Analysis (to aggregate several simple metrics into one meaningful combined metric).
6. Target setting.

A cross-work stream collaboration between c4c partners led to the selection of an initial core set of 13 metrics (Appendix B) from a list of 126 proposed metrics. The core set, prioritised by function and business case, is used to measure the performance of the studies used to define the network’s processes and to test its viability (so-called proof-of-viability studies), thereby testing the usefulness and actionability of this core set. Each metric has a target (value or range) and several attributes defined, including Name and Code, Process (mapped to Network or Clinical Trial processes), Definition, Data Points, as well as prioritisation for collection. The subset was chosen after a three-month consultation process across all c4c National Hubs and Industry partners of the consortium. The c4c Network Committee approved the metrics after a pilot phase of utilising with academic proof-of-viability studies. These metrics are critical to the c4c network and trial performance management framework and are continuously reviewed and evaluated.

#### MICYRN—Maternal Infant Child and Youth Research Network

In early 2019, MICYRN collaborated with I-ACT to learn about the PICTR initiative and metrics collected in the United States. Following the discussions with I-ACT, MICYRN engaged with its clinical trials consortium (CTC) comprised of scientific and operational representatives across 16 clinical trial units at MICYRN’s member research organizations to discuss the Quality Improvement (QI) and Performance Metrics initiative. Buy-in from the CTC was achieved and deemed important to the maternal-child health research community in Canada. The MICYRN leadership team conducted individual teleconferences with CTC sites to identify a list of meaningful indicators across the 3 domains of quality, efficiency, and timeliness; 11 interviews were completed. Using the interview data, an electronic survey was created with the compiled list of 14 indicators and disseminated to the 16 consortium sites for completion. Sites were asked to rank each indicator in order of importance to their site (1–14). 11/16 CTC sites completed the survey. The survey results were analysed, reducing the list to the top 6 indicators identified by the CTC sites. The 6 indicators were reviewed by the MICYRN leadership team and in terms of tangible action items that MICYRN could support and facilitate. The MICYRN Annual General Assembly brought together the CTC to collectively generate common data elements and definitions, inclusion/exclusion criteria, timeframe, methods of data collection, frequency of reporting, and unit analysis, further reducing the indicators to 5 (Appendix C). The CTC and MICYRN leadership team are currently working on metrics collection and action items for each of the 5 defined indicators.

In summary, metric selection was driven by site quality improvement in iACT (11 metrics), by network performance in c4c (13 metrics), and by both in MICYRN (5 metrics).

### Commonalities and Challenges in Identifying Network Metrics

Appendix A–D describe the metrics provided by the participating networks. The metrics developed are broadly at either trial level, or at site, and/or country level. They are related to individual services developed, and/or network/infrastructure

**Figure 1:**
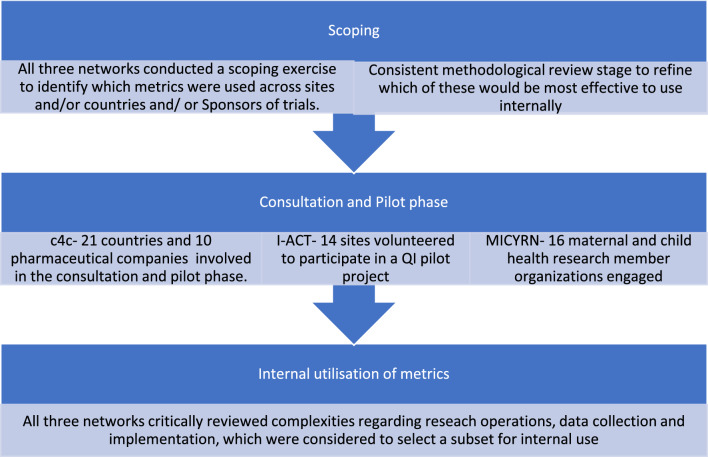
Commonalities of the approach to identifying and developing metrics across iACT, c4c and MICYRN.

Figure 1 summarizes the commonalities of the approach to identifying and developing these metrics across the three networks. All networks used a staged evidence-based approach based on existing evidence and wide internal stakeholder consultation and co-creation, keeping in mind the expected implementation of metrics across sites and organizations.

Appendix D summarises metrics related to each phase across each contributing network. The network driven by site quality improvement did not have indicators for capacity/capability or identification/feasibility (Table 1). 15 metrics for trial start up and conduct were identified. Metrics related to approvals were found in all three networks. Topics relating to protocol review were only included by the network driven by site quality improvement. Topics relating to numbers of paediatric interventional clinical trials and investigators participating in these at country level were only included by the network focussing on country-wide approach. Site identification/feasibility indicators were only included by the network that was driven only by network management.

**Table 1:** Common areas of trial lifecycle where metrics are used by all three contributing networks

Trial lifecycle and network activity domains	I-ACT	c4c	MYCIRN
Research capacity/capability indicators	**X**	1 metric	2 metrics
Site identification/feasibility indicators	**X**	2 metrics	**X**
Trial start up indicators	5 metrics	7 metrics	1 metric
Recruitment/enrolment indicators	6 metrics	3 metrics	2 metrics

The challenges faced when reviewing and identifying common metrics reported by the three networks were:

Technical differences: c4c, I-ACT and MICYRN use (and source data from organisations that may use) different technical standards and systems, making it difficult to exchange data and information.

Measurement and semantic differences: All three networks use different terminology, definitions for each data point and metrics, and coding systems, making it difficult to compare data across organizations. Each of the three networks used slightly different reference points and definitions to capture similar metrics. For example, specific definitions used for site “initiation”, “activation” and “ready for enrolment” timelines were different between networks, impacting how the dates for these steps were captured. The same was noted in recruitment dates related to patient screening, consent, or enrolment. The source of information also varies; c4c collects detailed information from sponsors, whereas I-ACT and MICYRN collect the information from sites.

Organizational policies: Parent and partner organisations have different policies and regulations regarding data sharing and use; these need to be addressed to establish common guidelines for data exchange. These differences often arise because of the characteristics of health systems. Our findings and discussion suggest that metrics are identified, defined, and developed according to each stakeholder's goals and the processes they can measure or influence.

### Working Towards a Common Interoperable Set of Metrics

By comparing the identified metrics across the networks, we found specific shared metrics measured across all three networks that can form the basis of comparators for the service/support that the networks provide across the trial lifecycle. Shared metrics could measure the effectiveness of interoperable networks. An example of a shared metric is shown in Table 2, illustrating challenges with terminologies and data point/measures alignment.

**Table 2:** An example of a common metric to demonstrate challenges with terminologies and data point/measures alignment

Outcome	Network	Metric/measure	Definition	Data point
**Site initiation/Site activation to First patient consented/enrolled/screened**	I-ACT	Time from site initiation visit to First patient consented	This measure counts the total number of calendar days from site initiation visit to the date the first patient is consented ***Site initiation visit:**** Visit conducted by the monitor to prepare and set up a research site to conduct a study* ***First patient consented:**** The date the first patient signed the study consent, whether the patient was enrolled or not*	• Date of site initiation visit • Date first patient consented
	c4c	Elapsed time from Site activation to Last patient enrolled	This c4c core set metric is a combined metric which contains a set of datapoints to calculate sub metrics, including elapsed time between site activation and first patient screened; last patient screened; first patient enrolled; and last patient enrolled The comparable sub metric definition for this outcome would be: Elapsed time between site activation and first patient screened ***Site activation****: Open for recruitment date (site level)* ***First patient screened*:** *Actual date first person screened*	• Open for recruitment date (site level) • Actual date First person screened • Actual date Last person screened • Actual date First person enrolled • Actual date Last Patient enrolled
	MICYRN	Time from Site Initiation to First Patient Enrolled	This measure assesses the number of calendar days elapsed between the date of site initiation (this will be unit specific, could be REB approval/contract execution/institutional approval) and the date of enrolment ***Site Initiation:**** Will be unit specific depending on the final approval needed before a trial can start (may be REB approval, contract execution, institutional approval, *etc*.)* ***Enrolled:**** signed informed consent form* + *first visit attended (this indicator will be based on intention to treat for analysis purposes)*	•Date of Site Initiation •Date First Patient Enrolled

## Discussion

The adoption of rigorous, harmonized operational metrics along with performance targets can support tracking, evaluating, benchmarking, and predicting performance [13, 14]. To our knowledge, this is the first report of international comparisons between international paediatric clinical research networks.

c4c, I-ACT and MICYRN each have developed and implemented a well-defined set of metrics. Despite differences, common ground exists in the approaches, methods and sources of data collection for these metrics. The review and usage of these metrics are defined by each network’s internal goals. The main aim aligned across all three networks is to ensure the efficient conduct of clinical trials across the network sites. Adopting common metrics, standards and terminologies across organizations helps ensure data interoperability, identifying common trends, and allows the networks to work towards benchmarking. The networks have worked together to identify a core set of metrics which is comparable to other multi-site, multi-national clinical research organizations working towards efficiency in trial set-up, enrolment, and completion [9].

Developing common metrics for multiple networks from the start of the networks would be ideal. Each network needs to establish its focus and identity before liaising with other networks. Several challenges exist concerning I-ACT, MICYRN and c4c using metrics inter-operably. Some of these challenges can be addressed by clearly identifying specific collaborative activities that address organisational, measurement and technical issues in a comprehensive and coherent manner. One challenge relates to the specific nature of what is being measured and how, which ultimately impacts the measurement properties, utility, and impact of selected metrics, pertinent to how the metrics and underlying data points were defined. The absence of a widely accepted standard for data nomenclature, exchange and interoperability means that theses aspects will need to be addressed within each network and then across the networks. For future inter-operability, all 3 networks will need to agree upon common sets of metrics and accompanying definitions.

Another general limitation of metrics-driven network initiatives is the oversight and influence that each network has over their respective sites. Each network has been designed and established with its partner organisations with differences in communication channels, sponsor interactions activities, and structures, all of which impact the information collection and flow through the organisations. The networks cannot mandate sharing of data because different partner organizations have different cultures, governance, and ways of working, which can impact their willingness and ability to collaborate on interoperability efforts. In particular, there is a limitation in capturing and interpreting variations in some metrics that are beyond the control of the network. An example of this is seen in recruitment timelines. The recruitment metrics were mostly aggregated and do not consider potential reasons for efficiency or delays. This limitation makes it difficult to identify specific actions for standardization and improvement in the future. Furthermore, c4c and I-ACT both have specific roles and objectives for their organizational sites or country-level networks and they receive a mixture of private and public funds to support the initiative. On the other hand, the sites affiliated with MICYRN are academic member organizations that operate without dedicated funds to support their metrics collection initiative. As a result, MICYRN does not have the same level of influence and incentive for their sites, and the collection of certain metrics largely depends on the individual sites rather than the network. This poses additional challenges in gathering accurate and comprehensive metrics.

Despite these challenges, the overarching goal of these networks is to improve the conduct of clinical research studies sponsored by industry and academia. Interoperable metrics will ensure that clinical research study operations across different networks can be reported on in a standardised manner, which makes it easier to compare data across different networks and countries. A more comprehensive, consistent and accurate view across sites and countries is possible, allowing better identification of issues and informed decisions to be made based on high-quality data. The above advantages will support tracking and performance management of network activities supporting clinical trials, collaborative decision making, and solution finding. Sharing of good practice and learnings across networks will result in efficiencies of trial set up and enrolment stages, thereby reducing costs and ultimately helping improve patient outcomes. Shared metrics and targets establish a common framework that will allow better identification of bottlenecks and hurdles in the trial delivery process, and development of quality improvement initiatives to support site and organizations, including adequate resourcing and process improvement.

Interoperable metrics enable clinical research networks to collaborate and share data, which can lead to increased efficiency and the development of new treatments and therapies. Other existing clinical research networks around the world that include paediatric research activities collect clinical trial research performance metrics or benchmark data to assess the performance of their sites. However, the methodologies for collecting such data vary. Without a universal standard or methodology in place, networks cannot reference the same metrics across all domains of the trial lifecycle. For example, based on publically available data, the Paediatric Trials Network (PTN) addresses a reduction in time from the start of a study to completion and increased enrolment as part of its organizational improvements to increase efficiency [19] and the Cystic Fibrosis Foundation (CF) Benchmarks [20] use metrics focused on time to contract execution and time to first patient inclusion, whilst metrics from other networks such as the CTSA- Clinical Research Consortium and the Pediatric Emergency Care Applied Research Network (PECARN) [21] do not address these areas of activity. Conversely, even when many networks focus on the same domains, methodological differences in the approach can be identified. That would be the case when contrasting metrics focused on time to IRB approval and recruitment from the three aforementioned research networks. These disparities are not dissimilar to the ones we identified, and can be at least partially justified by different contexts and purposes from each network, as well as constraints to data sources and data collection.

Efforts are being made to establish more standardized approaches to data collection and measurement in clinical research [14]. Regulatory bodies, professional organizations, and research institutions are working towards developing common frameworks and guidelines to facilitate the collection and reporting of data across different sites and networks. These initiatives aim to promote consistency and comparability in performance metrics, which can lead to better assessment and evaluation of clinical trial outcomes. It is important for clinical research networks and stakeholders to collaborate, share their experiences and knowledge to establish common methodologies and definitions to help advance the field and ensure the rigorous conduct of clinical trials, ultimately benefiting patients and advancing medical knowledge.

It should be noted that this paper only focuses on a small sample of industry- and academia-facing large paediatric trial networks that are specialty agnostic with wide geographical coverage. Other networks focused on specific disciplines or covering other study designs may require a tailored approach to the selection of relevant operations metrics. In addition to these specific metric related limitations, interoperability efforts require significant funding and resources, as well as time and commitment to work together, which may not be available to all organisations.

### Recommendations and Next Steps

The think-tank identified specific collaborative activities that are needed to develop and use interoperable metrics:

1. Harmonization of processes for the collection of data related to metrics, including goals, data definitions, and measurement methods, across organizations can help ensure data comparability.
2. Collaborative development of technology solutions that support interoperability, such as common data platforms and APIs.
3. Provision of education and training to staff on the importance of data requirements, capture and integrity. Shared educational and training opportunities focusing on quality improvement may reduce burden on resources.
4. Work with similar networks, e.g. those that may be academia-facing or not mandated to study new drugs with industry [22], on interoperable metrics and their implementation
5. Addressing context at site and national level and regularly testing and evaluating the interoperability of data and systems across organizations to help identify and resolve any issues.
6. Engaging stakeholders, including patients, healthcare providers, and regulatory agencies, in the interoperability efforts so that the solutions developed meet their needs and are widely adopted.
7. Developing a multi-stakeholder engagement strategy for sites to be involved in metrics projects and across the three networks would further ensure interoperability.
8. Establishing data sharing agreements to ensure the secure exchange of data and information.

The think tank proposes the following next steps to utilise these metrics inter-operably:

1. Define common or similar metrics/terminology that can be used within each network (intra network metrics) but be alike enough to allow interpretation as globally aligned networks.
2. Define and align on data points that are collected to measure each metric.
3. Review target values and/or comparators and/or benchmarks that may be used to drive performance.

## Conclusion

This paper presents a review on the experience of three international paediatric clinical research networks to establish metrics for paediatric clinical trial support, demonstrating a disparity in methodology and common challenges in defining metrics. The adoption of rigorous, validated, and harmonized operational metrics, along with performance targets, can bring several advantages to international paediatric research networks. The recommended next steps will contribute to enabling international collaboration and benchmarking, thereby resulting in more efficient trial set-up, enrolment, and completion, reducing costs and improving patient outcomes.

## Data Availability

Not applicable.

## Appendix A: I-ACT (*PICTR)*—11 Site Operation Metrics

**Table:**

1	Time from receipt of study start-up packet to first patient consented
2	Time from receipt of study start-up packet to site initiation visit
3	Time from receipt of budget to final budget approval
4	Time from receipt of contract to final contract approval
5	Time from first ancillary protocol review to final ancillary protocol review
6	Time from submission of initial IRB application to final IRB approval
7	Time from site initiation visit to first patient consented
8	Accrual ratio
9	Time to fully enrol patients in a study
10	Percentage of targeted enrolment goal met
11	Percentage of patients completing study

## Appendix B: c4c Core Set of 13 Metrics for Network Management (from a Longer List of 126 Metrics)

**Table:**

1	Elapsed time for the site to complete the study specific feasibility assessment questionnaire
2	Elapsed time for c4c to complete the site form from when request sent by the Single Point of Contact
3	Elapsed time for signing the Confidentiality Disclosure Agreements between Sponsor/Single Point of Contact/National Hub/Site
4	Number of sites activated (opened) on time
5	Elapsed time from site selection to site activation
6	Elapsed time between protocol approval to first site activated
7	Elapsed time between contract signed and site activation
8	Elapsed time between contract submission and contract signing date by the site
9	Elapsed time between submission of a c4c reviewed protocol to the Independent Ethics Committee (IEC) and approval of the protocol by the IEC
10	Elapsed time between final review of protocol by c4c and National Competent Authority submission
11	Number of sites achieving agreed recruitment targets
12	Number of patients screened but failed to enrol
13	Elapsed time from site activation to last patient enrolled

## Appendix C: MICYRN 5 Key Indicators Identified for Metrics and Performance Initiative

**Table:**

1	Number of investigators participating in paediatric research
2	Number of interventional clinical trials in child health research
3	Time from site initiation to first patient enrolled
4	Percentage of targeted patients enrolled
5	Time for study start up

## Appendix D: Detailed List of the Specific Metrics Related to Each Phase Across Each Contributing Network

**Table:**

Trial lifecycle and network activity domains	I-ACT	c4c	MICYRN
Indicative indicators of research capacity/capability		Elapsed time for c4c to complete site form from when request sent by the Single Point of Contact	Number of child health investigators participating in interventional clinical trials in Canada Number of regulated child health, interventional clinical trials conducted in Canada
Site identification/feasibility indicators		Elapsed time for the site to complete the study specific feasibility assessment questionnaire Elapsed time for signing the Confidentiality Disclosure Agreements between Sponsor/Single Point of Contact/National Hub/Site	
Trial Start up indicators	Time from receipt of budget to final budget approval Time from receipt of contract to final contract approval Time from first ancillary protocol review to final ancillary protocol review Time from submission of initial IRB application to final IRB approval Time from receipt of study start-up packet to site initiation visit	Elapsed time between contract submission and contract signing date by the site Elapsed time between submission of a c4c reviewed protocol to the Independent Ethics Committee (IEC) and approval of the protocol by the IEC Elapsed time between final review of protocol by c4c and National Competent Authority submission Elapsed time between protocol approval to first site activated Elapsed time from site selection to site activation Elapsed time between contract signed and site activation Number of sites activated (opened) on time	Study start up time
Recruitment/Enrolment indicators	Time from receipt of study start-up packet from first patient consented Time from site initiation visit to first patient consented Accrual ratio Time to fully enrol patients in a study Percentage of targeted enrolment goal met Percentage of patients completing study	Number of patients screened but failed to enrol Elapsed time from site activation to last patient enrolled Number of sites achieving agreed recruitment targets	Time from site initiation to first patient enrolled Percentage of targeted participants enrolled
